# Therapeutic application of adipose-derived stromal vascular fraction in diabetic foot

**DOI:** 10.1186/s13287-020-01825-1

**Published:** 2020-09-14

**Authors:** Xiansheng Zhao, Jiamin Guo, Fangfang Zhang, Jue Zhang, Delin Liu, Wenjun Hu, Han Yin, Liang Jin

**Affiliations:** 1 School of Life Science and Technology, China Pharmaceutical University, Nanjing, 211198 Jiangsu Province China; 2 Irell & Manella Graduate School of Biological Sciences, City of Hope National Medical Center, California, 91010 USA; 3 Department of Endocrinology, The Affiliated ZhongDa Hospital of Southeast University, Nanjing, 210009 Jiangsu Province China

**Keywords:** Diabetic foot, Stem cells therapy, Stromal vascular fraction (SVF), Cytokine, Vascular reconstruction

## Abstract

Diabetic foot is one of the severest complications of diabetes. In severe cases, this disease may be lead to amputation or even death due to secondary infection and ischemic necrosis. Since the ineffectiveness of traditional therapy, autologous stem cell transplantation has been used to treat diabetic foot. This simple, safe, and effective therapy is expected to be applied and promoted in the future.

In this review, we described the detailed pathogenesis of diabetic foot and the common clinical treatments currently used. We also revealed vascular remodeling as the potential mechanism of therapeutic functions of adipose-derived stromal vascular fraction (SVF) in treating diabetic foot.

## Background

Recently, the incidence of diabetes is increasing in the world, and the age of onset has decreased year by year. Diabetes has become a health problem that cannot be ignored and the prolonged presence of hyperglycemia could induce various complications including diabetic foot [1].

Diabetic foot is caused by diseases of peripheral blood vessels at different degrees and abnormalities of the distal lower limb nerves in diabetic patients, resulting in foot infections, ulcers, and deep tissue damage [2]. Peripheral vascular disease of diabetes often involves the lower extremity arteries. At the early stage, it is mainly characterized by intermittent claudication. As the arterial stenosis increases, the patients could have rest pain or even could not walk. Later, the ischemia with infection and neuropathy could cause local tissue necrosis, ulcers, and gangrene, leading to ischemic diabetic foot [3]. Finally, 10% of patients’ toes have to be amputated, and the serious infection caused by diabetic foot might even threaten their lives [4]. It has been reported that infection is the main cause of disability and death in diabetic foot patients, while neurogenic and ischemic infections account for 45 ~ 60%, of which 25 ~ 44% are caused by the neuropathy, and 10% are caused by blood-lacking [5]. This disease affects the patients’ life quality and brings a heavy economic burden to their families.

Since the pathological process of diabetic foot is extremely complicated, traditional medical therapy, vascular bypass, interventional surgery, and amputation treatment methods have certain limitations [6]. Stem cell transplantation technology for the treatment of diabetic foot is a hotspot of transplanted angiogenesis in recent years. Stem cells have the characteristics of high self-renewal, proliferation, and multiple differentiation potential, as well as paracrine effects, which promote the regeneration of blood vessels and nerve tissues around the transplant site so that the affected ulcers could achieve blood circulation reconstruction and blood supply improvement and, eventually, achieve the purpose of healing diabetic foot [7–9].

The stromal vascular fraction is an active component in adipose tissue and contains a variety of cells including adipose stem cells [10]. Since the various cell types, they have strong regenerative potentials such as angiogenesis, tissue remodeling, and immune regulation. It has been used for clinic treatment of various conditions and shown good safety and healing effects. Moon and colleagues injected SVF cells into the affected area of diabetic foot patients, and the microvascular blood flow of the wound surface was found significantly increased with no adverse reactions [11, 12]. Studies found that SVF could secrete a variety of cytokines, differentiate into various cells such as endothelial cells, and have the potential to participate in angiogenesis in vivo*.* Therefore, it can improve local blood circulation and form the microenvironment that is conducive to tissue cell survival and functional maintenance [13–15].

## Pathogenesis of diabetic foot

### Diabetic foot caused by neuropathy

The most common complication of diabetes is diabetic neuropathy, accounting for 30% to 50% of diabetic patients. The incidence is similar for men and women, but men usually occur earlier than women as their lifestyle and testosterone deficiency [16]. The symptoms of diabetic neuropathy include central neuropathy and peripheral neuropathy, resulting in sensory, motor, and autonomic neuropathy. The pathogenesis of the disease may be related to the increase of neurotrophic factors and the decrease or disappearance of protective factors [17]. Sensory neuropathy is an important cause of diabetic foot. It often involves the small nerves that innervate the pain and temperature of the foot, which leads to a decrease in the sensitivity of the patient to stress-related trauma and skin damage. Therefore, the self-protection mechanism is lost, and the harmful stimulation cannot be sensed in time so that the foot is vulnerable to injury [18]. Motor neuropathy mainly affects small peripheral nerves, which lead to abnormal innervation of the gastrocnemius muscle group (mainly flexor) in feet. This imbalance in innervation will cause a change in the shape of the foot, causing the humeral head to protrude. Meanwhile, under the friction of improper footwear and gravity distribution, it is easy to cause foot damage, which leads to foot deformity and ulcer [19]. Autonomic neuropathy is the most complicated in the diabetic neuropathy, which could involve all organs and systems of the whole body. After the occurrence of autonomic neuropathy, the ability of autonomic regulation of skin blood flow is lost. At the same time, the arteriovenous short circuit and shunt increase. Although the skin temperature is not low and the color is pink, the nutritional blood supply has decreased [20]. Also, autonomic neuropathy could cause a lack of secretion of sweat and lipid glands, which makes the skin dry, prone to chapped, and become the gateway to bacterial invasion, eventually leading to ulcers [21].

### Diabetic foot caused by vascular lesion

Diabetes can cause a wide range of vascular diseases, involving arteries, veins, and capillaries. Vascular disease leads to the decrease or even occlusion of blood flow in lower limbs, which becomes the pathological basis of diabetic foot [22]. The major vascular disease caused by diabetes is mainly atherosclerosis. The main causes may include hyperlipidemia, hyperglycemia, carbon monoxide reduction, insulin resistance, hemagglutination abnormalities, changes in blood flow, etc. [23]. When atherosclerosis occurs in diabetic patients, the plaque contains more calcium, the expression of inflammatory markers increases, and it affects the blood vessels in the more distal part, especially the foot. It is more difficult to form the collateral circulation after vascular occlusion [24]. The risk of diabetic foot ulcer increased significantly after diabetic peripheral arterial disease, and often asymptomatic until the ulcer occurred. Besides, the reduction of blood supply is not only conducive to wound healing and response to infection, but also more likely to make foot lesions [25].

The microvascular complications of diabetes are mainly microcirculatory disorders, including microvascular disease, microvascular disorder, and changes in blood physicochemical properties. In the pathogenesis of diabetic foot, these three interact with each other and cause each other [26]. Diabetes may cause changes in microvascular function, oxygen partial pressure, and vascular permeability, as well as disturbance of rhythmic contraction of arterioles and arterioles. These abnormalities can cause a decrease in capillary blood flow in the skin and an increase in vascular permeability and platelet aggregation, which leads to the thickening of vascular endothelial cells and aggravation of microvascular disease. After a long time, it could cause vascular stenosis, insufficient perfusion of tissues and organs, and blood circulation disorder at the distal end of the limb, which eventually leads to the occurrence of diabetic foot [27].

### Diabetic foot caused by infection

If peripheral neuropathy and vascular lesion are indispensable conditions for the development of the diabetic foot, infection is the direct reason for diabetic foot [28]. The abnormal immune response of diabetic patients leads to the impairment of the phagocytic capacity of neutrophils, which weakens their response to infection or injury. At the same time, ischemia will further aggravate this response [5]. Foot infection generally could be attributed to four independent risk factors: deep trauma, recurrent trauma, long-lasting trauma (> 30 days), and peripheral vascular disease [29]. About one-third of diabetic patients develop a fever when they have a foot infection, but the number of white blood cells may not increase despite widespread infection. However, if the patient has sensory neuropathy, the perception of pain or temperature is weakened, so the perception of infection will be delayed [30]. If the patient’s blood sugar cannot be controlled, the infection will be difficult to control. Also, the blood circulation disorder will eventually lead to the rapid deterioration of the wound [31] (Table 1).

**Table 1:** Pathology of diabetic foot and the main citation

Pathology of diabetic foot			Citations
Neuropathy	Sensory neuropathy	Reduced sensitivity	[17] [18]
	Motor neuropathy	Foot deformity	[19]
	Autonomic neuropathy	Flow-mediated disorder	[21]
Vascular lesion	Macroangiopathy	Atherosclerosis	[22] [23]
	Microangiopathy	Microcirculatory disorders	[26]
Infection	Main incentive	Accelerate wound deterioration	[28]

## Current therapy for diabetic foot

Current treatment for diabetic foot includes drug therapy, vascular intervention therapy, dressing adjuvant therapy, hyperbaric oxygen therapy, vacuum suction therapy, growth factor therapy, and stem cell therapy [32].

However, none of the above are sufficient for treating diabetic foot. Drugs used in diabetic foot are mainly for vascular diseases, neuropathy, and hypoglycemic drugs [33]. Drug therapy for patients with mild and moderate diabetic feet simply delays the development of their lesions; it is the basis for the treatment of diabetic foot. Once the drug therapy is not effective and the ischemia is getting serious, the vascular intervention surgery needs to be used to reconstruct the blood flow [34]. However, since most of the diabetics are old and frail, who often suffer from cardiovascular and cerebrovascular diseases, thus, the patients cannot tolerate the stimulation of surgical bypass. Besides, the vascular lesions involve many small arteries and segments, which leads to the lack of distal artery outflow channel in some patients, and thus, the vascular bypass graft and interventional treatment are not applicable [35]. Meanwhile, long-term follow-up showed that patients with diabetic foot were prone to restenosis after vascular intervention. The proportion of patients who opt for amputation remains high [6].

Also, non-surgical therapies include dressing adjuvant therapy, hyperbaric oxygen therapy, and vacuum suction therapy. This kind of simple physical auxiliary intervention could improve the wound inflammation and microcirculation, which promote wound healing, but the treatment requires long-term hospitalization while the effect is not that significant [36]. Cytokine treatment to promote wound healing is a new method for diabetic foot. The cytokines used mainly include fibroblast growth factor (FGF), epidermal growth factor (EGF), platelet growth factor (PGF), vascular endothelial cell growth factor (VEGF), cell colony-stimulating factor(G-CSF), and hepatocyte growth factor (HGF) [37].

In recent years, stem cell transplantation has been considering a cutting-edge technology to treat diabetic foot. Although stem cells are not currently used as a routine means for treating diabetic foot in clinical practice, many researchers have reported that stem cell transplantation could promote neovascularization of ischemic limbs, improve and restore limb blood flow, and ultimately cure the diabetic foot [38–41].

## Therapy for diabetic foot with stem cells

Stem cells are a class of cells with self-renewal and multi-directional differentiation potential that can differentiate into specific types of cells under specific conditions [42]. Stem cell transplantation for the treatment of diabetic foot is mainly based on the principle that stem cells can differentiate into vascular endothelial cells and smooth muscle cells in vivo, secreting numerous pro-angiogenic factors to form new blood vessels. The stem cells are transplanted into the ischemic lower limbs to gradually differentiate and form new capillaries, which improves and restores blood flow to the lower limbs, and achieves the purpose of treating diabetic foot [43].

Currently, the stem cells used to treat diabetic foot are derived from many tissue sources such as the bone marrow, umbilical cord, placenta, cord blood, heart, liver, spleen, pulp, dermis, and adipose tissue. Among them, adipose-derived stem cells are advantageous of the widely distributed but low immunogenicity adipose tissue, which is easier to obtain but less harmful to patients [44]. Many initial studies were adipose-derived stem/stromal cells (ASCs) identified by Zuk, which have received extensive attention due to their pluripotent differentiation potential, paracrine properties, and significant influence on regenerative medicine [45]. ASCs are a group of homogeneous stem cells obtained by the adherent subculture of freshly isolated SVF. SVF is a heterogeneous cell population obtained by digesting adipose tissue [46]. However, compared to ASCs, SVF does not require any cell culture process and can be used directly after separation. Therefore, it is relatively safer and can meet lower regulatory standards, which is favored by many clinical researchers [47].

SVF is widely used to improve diabetic foot [48]. Tan’s research found that SVF can increase the viability and migration of fibroblasts in a hyperglycemic environment by upregulating cytokines around the wound, thereby accelerating wound healing [49]. Chae used SVF to treat diabetic foot and found that not only the number of fibroblasts increased significantly, but also the amount of collagen synthesized by fibroblasts increased significantly. The results of subsequent clinical experiments showed that when 100% of the wounds of patients treated with SVF were healed, the healing rate of the control group was only 62%, indicating that SVF could accelerate wound healing [50] (Table 2).

**Table 2:** Diabetic foot treatment and the therapy features

Treatment of diabetic foot		Features	Citations
Therapy	Drug therapy	For mild and moderate patients; just delay the course; basic therapy	[33]
	Vascular intervention therapy	Suitable for severe ischemia; except for elderly and weak patients	[34]
	Dressing adjuvant therapy	Non-surgical therapy; physically assisted intervention; poor effect	[32]
	Hyperbaric oxygen therapy		[36]
	Vacuum suction therapy		[36]
	Growth factor therapy	Use multiple cytokines; good effect but expensive; new therapy	[37]
	Stem cell therapy	Significant treatment effect; wide range of applications; cutting-edge technology	[38, 40]

## SVF cells for diabetic foot

### Acquisition of SVF cells

The adipose tissue in the human body is generally divided into subcutaneous fat and visceral fat, which can be obtained by liposuction or surgical resection. Common materials include abdomen, buttocks, forearms, groin, etc. [51]. In general, the separation methods of SVF could be generally divided into two categories: enzymatic methods that use proteolytic enzymes to digest adipose tissue and physical and mechanical processing methods that do not use proteolytic enzymes [52]. Enzymatic methods often use type I collagenase to digest adipose tissue. The general process is summarized as follows: the adipose tissue and collagenase are mixed at an appropriate ratio and placed in a 37 °C environment for 1 h with shaking and digestion; then an equal amount of complete medium is added to terminate the digestion. The supernatant is discarded by centrifugation, then washed with phosphate buffer saline (PBS), and finally, filtered through a specific pore size filter to obtain an SVF suspension. Physical and mechanical digestion of adipose tissue, including methods such as serum digestion, mechanical shaking, and bolus shearing. Compared to enzymatic methods, this kind of digestion method takes more time, and the cell yield and activity are not good enough; thus, it is not widely used [53].

### Cell populations in SVF

Human adipose tissue can be easily obtained from the abdomen, buttocks, forearm, or groin liposuction or surgical resection. SVF cells could be obtained after adipose tissue is digested with proteolytic enzymes [54]. SVF cells isolated from the adipose tissue are heterogeneous cell populations containing a variety of cells. This multicellular component could be identified by different cell surface molecules. Surface molecules of cells, the cluster of differentiation (CD), could be used to identify different cell types in a cell population [55]. The International Cell Therapy Association has stated to define SVF using this identification method. At present, the academic community has reached a consensus on the cell types contained in SVF cells [56]. SVF cells contain a variety of cell types, such as ASCs; hematopoietic stem cells (HPCs), mesenchymal cells; and endothelial progenitor cells (EPCs), endothelial cells, pericytes, and macrophages [57–59].

### Application of SVF in diabetic foot

SVF cells are able to promote the recovery of the diabetic foot through various effects. SVF, for instance, contains various cells which directly or indirectly secrete bioactive factors to induce degradation of the basement membrane, affecting the proliferation and migration of endothelial cells, thereby promoting the fusion and remodeling of new blood vessels. Also, these cytokines could increase the stability of the pericytes to the vascular network [13, 49, 60].

#### Differentiation of SVF

Among SVF cells, ASCs not only occupy a relatively large amount of cells but also have strong differentiation ability. ASCs are pluripotent stem cells that could differentiate directly into vascular endothelial cells, smooth muscle cells, and pericytes. These cells regulate vascular growth, stabilization, and maturation through activation of TGF-β, angiopoietin-2, PDGF-B/PDGFR-β, Notch, and S1P/Edg signaling pathways [61, 62]. Meanwhile, pericytes not only promote the appearance of endothelial progenitor cells but also maintain vascular integrity to form a vascular network [63]. Studies have shown that ASCs could also participate in the formation of new micro-vessel together with endothelial cells to form a stable vascular network system [64]. Animal experiments have demonstrated that transplanted ASCs could differentiate into endothelial cells, significantly improving blood flow and capillary density in diabetic and non-diabetic animal models of lower limb ischemia [65].

#### Paracrine action of SVF

Studies have found that when SVF cells are transplanted into the ischemic area, their vascular density, blood flow, and secreted hepatocyte growth factor, vascular endothelial growth factor, and basic fibroblast growth factor (bFGF) are significantly increased compared with the control group [66]. If the synthesis of HGF is inhibited, the ability of SVF cells to promote vascularization of ischemic tissue is significantly reduced [67]. Stem cells treated with VEGF antibodies also lose pro-angiogenic capacity in ischemic tissues [68].

ASCs in SVF cells could effectively secrete a large number of pro-angiogenic and anti-apoptotic factors, such as HGF, bFGF, VEGF, PGF-B, and TGF-β [69]. Prochzka isolated the factors secreted by ASCs and injected them into the ischemic limbs of rabbits. It was found that the blood perfusion of ischemic tissue in the experimental group was twice as high as that in the control group. Immunohistochemistry showed that the capillary density of the experimental group was significantly higher than the control group, which indicates ASC-secreted cytokines could promote angiogenesis [70].

In addition to ASCs, other components in SVF cells can also promote vascular remodeling through the paracrine pathway. Studies have shown that hypoxia can induce macrophages to secrete vascular regenerative factors such as VEGF and bFGF, thereby promoting the formation of new blood vessels [71]. Macrophages in adipose tissues can be divided into M1 type macrophages and M2 type macrophages according to their activation states. In SVF cells, more than 90% of macrophages are M2 type [72]. M2 type macrophage is an anti-inflammatory macrophage. It can secrete anti-inflammatory factors such as IL-4, IL-10, TGF-β, and pro-angiogenic factors such as bFGF and VEGF, thereby inhibiting the inflammatory response and promoting vascular network formation [73].

Endothelial cells secrete exosomes, and adjacent endothelial cells can act as target cells to bind to exosomes, promoting endothelial growth, migration, and neovascularization [74]. Endothelial cells can activate the ERK1/2 signaling pathway by expressing CXCL-1, induce the secretion of epidermal growth factor, and promote angiogenesis [75]. Besides, stromal cells, fibroblasts, and smooth muscle cells can secrete HGF and regulate angiogenesis [76].

#### Mechanism of cytokine promoting vascular reconstruction

SVF cell components or hypoxic tissue can secrete a large amount of cytokines through paracrine, and these active substances can accelerate the healing of diabetic foot by promoting the biological function of the body cells [69–71]. For instance, VEGF could activate endothelial progenitor cells and induce endothelial cells to secrete a variety of cathepsins for degradation of extracellular matrix, as well as inhibit endothelial cell apoptosis and promote endothelial cell proliferation, migration, and neovascularization [77]. HGF binds to its receptor and promotes the proliferation of vascular endothelial cells by activating the Grb2/Sos-Ras-Raf-MAPK signaling pathway [76]. bFGF induces VEGF expression via the FGFR1/c-Src/p38/NFB-κB signaling pathway [78]. At the same time, the activation of NFB-κB can promote endothelial cell DNA synthesis, cell division, and cell proliferation and promote the regeneration of blood vessels [79]. TGF-β contributes to the production of extracellular matrix and promotes the interaction between endothelial cells and parietal cells, which in turn contributes to the formation of blood vessels [80] (Table 3).

**Table 3:** SVF cells for diabetic foot and its pros and cons

SVF cells for diabetic foot		Pros and cons	Citations
Separation methods	Enzymatic digestion	Simple and convenient; possible immune response	[52]
			[53]
	Physical method	Safety; long digestion time; low cell yield	
Cell populations	Multi-cellular components	Wide source; without culture; safety; effective	[57]
Mechanism	Differentiation	Direct action; directly involved in vascular reconstruction	[61]
	Paracrine action	Secrete various cytokines; promote vascular remodeling	[66, 69, 71]
	Cytokine action	Cytokine interaction; promote cell proliferation and migration	[77]

## Conclusion and prospect

As a new therapy for the treatment of diabetic foot, autologous adipose-derived stem cell transplantation has been widely used in clinical practice and has made it possible to exempt some patients from amputation or reduce the amputation plane and improve the quality of life, which fully demonstrates the feasibility of this technology [15, 81]. SVF is a heterogeneous cell population, which interact with each other to synergize with the whole process of the angiogenesis. However, the specific mechanism of synergistic angiogenesis and reconstruction between components still needs further investigation. Although SVF cells have achieved good therapeutic effects in many studies, further research is still needed to determine whether the cell therapy has side effects such as transplant toxicity and adverse reactions in the long-term treatment. The evaluation of the effect of SVF cells on the treatment of diabetic foot, including the survival of stem cells, the rate of proliferation and transformation, and the determination of the characteristics of transformed cells, still needs to be studied and solved in the future study.

In general, although using SVF cells to treat diabetic foot is still in the early research stage, it has enormous potential in clinical application. It is believed that with further investigation, adipose-derived SVF cells will ultimately show their unique cellular advantages and therapeutic value in clinic.

## Data Availability

Not applicable

## Abbreviations

SVF: Stromal vascular fraction
FGF: Fibroblast growth factor
EGF: Epidermal growth factor
PGF: Platelet growth factor
VEGF: Vascular endothelial cell growth factor
G-CSF: Granulocyte colony-stimulating factor
HGF: Hepatocyte growth factor
ASCs: Adipose-derived stem/stromal cells
PBS: Phosphate buffer saline
CD: Cluster of differentiation
HPCs: Hematopoietic stem cells
EPCs: Endothelial progenitor cells
bFGF: Basic fibroblast growth factor
